# Association of induced abortion with hypertensive disorders of pregnancy risk among nulliparous women in China: a prospective cohort study

**DOI:** 10.1038/s41598-020-61827-0

**Published:** 2020-03-20

**Authors:** Yinhua Su, Xiaoping Xie, Yanfang Zhou, Hong Lin, Yamei Li, Na Feng, Jiayou Luo

**Affiliations:** 1 Department of Maternal and Children Health, Xiangya School of Public Health, Central South University, Changsha, China; 2 Department of Community Nursing, School of Nursing, University of South China, Hengyang, China; 3 First Affiliated Hospital of University of South China, Hengyang, China

**Keywords:** Pre-eclampsia, Risk factors

## Abstract

The relationship between induced abortion(IA) and hypertensive disorders of pregnancy(HDP) is inconclusive. Few studies have been conducted in China. In order to clarify the association between previous IA and risk of HDP, including gestational hypertension(GH) and pre-eclampsia(PE), we performed a community-based prospective cohort study enrolling 5191 eligible nulliparous women in selected 2 districts and 11 towns of Liuyang from 2013 to 2015. Multivariable logistic regression was conducted to examine whether IA was associated with HDP, GH and PE. Of the gravidea, 1378(26.5%) had a previous IA and 258(5.0%) diagnosed with HDP, including 141(2.7%) GH and 117(2.3%) PE. The difference in the incidence of GH and PE between gravidae having one versus those with two or more IAs was minimal. After adjustment for maternal age, body mass index at first antenatal visit, education, virus infection and history of medical disorders, previous IA was significantly associated with HDP (OR = 0.67, 95%CI = 0.49 to 0.91) and PE (OR = 0.61, 95%CI = 0.38 to 0.97), but not with GH (OR = 0.73, 95%CI = 0.49 to 1.10). Additional adjustment for occupation, living area, anemia, gestational diabetes mellitus, psychological stress, conception climate and infant sex, multivariable analysis provided similar results. In conclusion, previous IA was associated with a lower risk of PE among nulliparous women.

## Introduction

Hypertensive disorders of pregnancy(HDP)is a pregnancy-specific disorder, including gestational hypertension (GH) and pre-eclampsia (PE). HDP complicated about 5.2%-8.2% of pregnancies and is still a major cause of maternal morbidity and mortality worldwide^1–3^. Furthermore, epidemiological studies have revealed that HDP is positively associated with the risk of future chronic disease, for example, heart failure, dysrhythmia, stroke, hypertension, diabetes mellitus(DM), end-stage renal failure etc^1^. Although significant efforts have been made, the causal mechanisms of HDP remain incompletely understood.

Previous studies have suggested that HDP is a primiparous disease, while a previous birth confers a protective effect on HDP^4–7^. This protective effect may attribute to immune tolerance to paternal antigen in fetal cells, and also to improve trophoblastic invasion after modification of maternal spiral arteries during the previous pregnancy^8^. However, whether induced abortion (IA), before which the woman has also been exposed to fetal cells, would offer similar protection against HDP in primiparous women is controversial. Some studies have shown that previous IA is protective against PE^6,7,9–11^. Nevertheless, no reduction in the incidence of PE, GH or HDP was also reported following previous IA^12–18^. At the other end of the spectrum, an IA in a first pregnancy was associated with a higher subsequent risk of PE^19^. In addition, most previous studies have been done in developed country, like UK^6,12–15,19^, USA^7,9,16^, Norway^10^, Finland^11,18^, New Zealand and Australia^15^. To our best knowledge, few community-based cohort study in China has been reported.

The incidence of HDP varies between different races^20,21^ and the prevalence of IA also varies in different countries and areas^22^. In China, approximately 10 million legal IAs equal to one-fifth of all abortions worldwide are performed annually^23^. the findings from previous studies may not be extended to Chinese population. Thus, considering the controversies above and the lack of studies in China, we carried out a community-based prospective cohort in Liuyang, Hunan, China, to evaluate whether IA is associated with a lower risk of HDP in the subsequent pregnancy.

## Results

### Study participants

**Figure 1:**
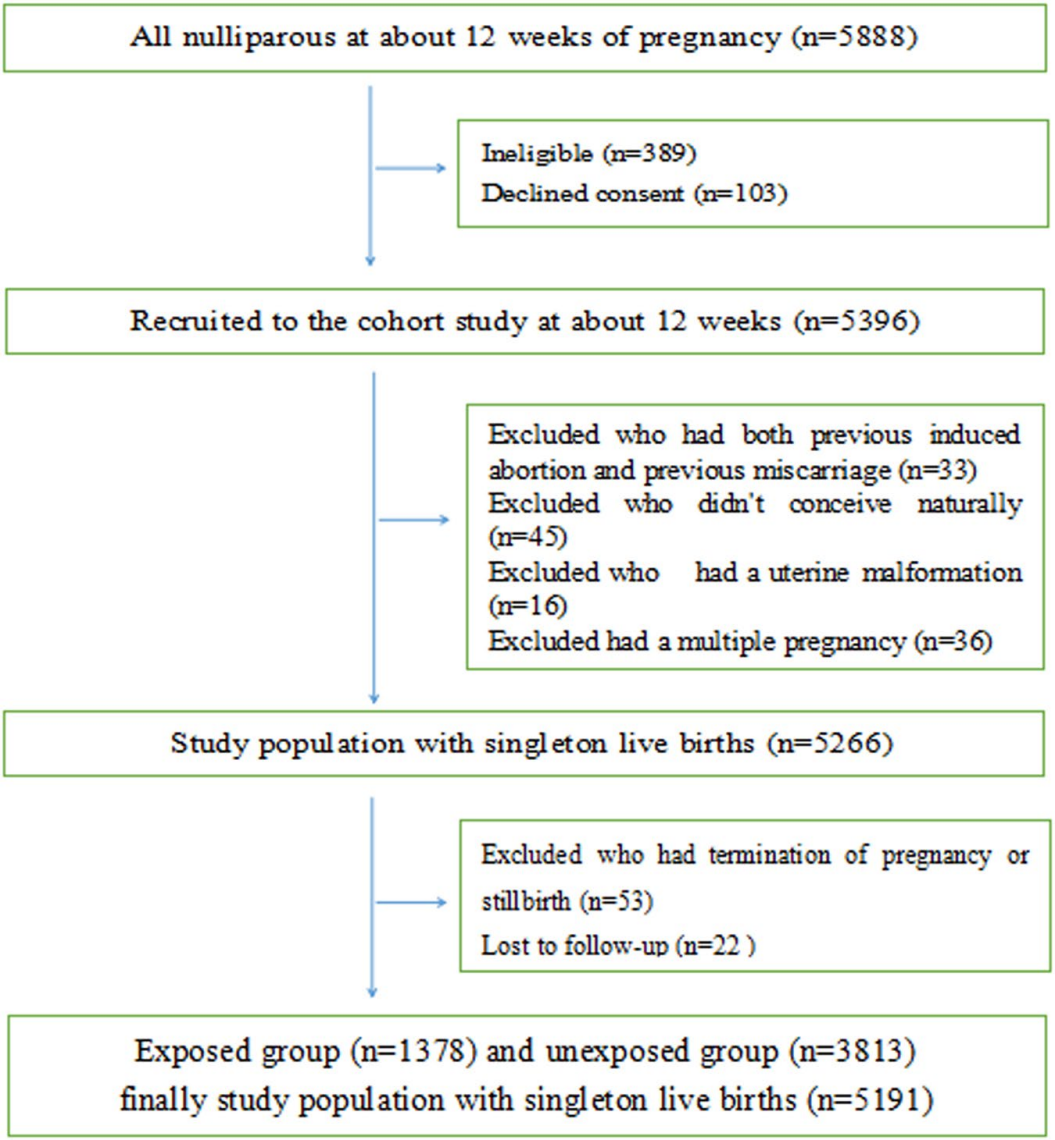
Recruitment of participants.

In the period June 2013 to November 2014, 5396 nulliparous women were recruited. Of these women, 33 (0.61%) had both previous IA and previous spontaneous abortion, 45 (0.83%) didn’t conceive naturally, 16(0.30%) had a uterine malformation and 36(0.67%) had a multiple pregnancy. Therefore, all these met the exclusion criteria. Follow-up to November 2015, 53(0.98%) had a terminated pregnancy or stillbirth, and 22 (0.41%) lost to follow-up for moving this area. Finally, we analyzed 5191(96.20%) nulliparous (Fig. 1).

### Maternal characteristics of the study participants

Of the 5191 nulliparous women, 1378 (26.5%) gravidae had one or more previous IAs (Table 1). Compare to none IA group, those women had previous IAs were more likely to be 35 years of age or older, educate less than 12 years, have BMI at first antenatal visit over 24 kg/m^2^, smoke, have virus infection and history of medical disorders(P < 0.05). Nonetheless, there were no significant differences in the distribution of occupation, living area, alcohol consumption, polyhydramnios, anemia, gestational diabetes mellitus(GDM), conception climate, infant sex and psychological stress between the IA group and none IA group.

**Table 1:** Distribution of selected characteristics of nulliparous women with and without induced abortion.

Characteristic		Observed population(N)	Previous IA		χ^2^	*p*
			No (N = 3813) (%)	Yes N = 1378 (%)		
Maternal age (years)	<35	5138	3789(99.4)	1349(97.9)	21.793	0.000
	≥35	53	24(0.6)	29(2.1)		
Education (years)	≤12	3974	2872(75.3)	1102(80.0)	12.193	0.000
	>12	1217	941(24.7)	276(20.0)		
Occupation	Unemployed	3091	2267(59.5)	824(59.8)	0.049	0.824
	Employed	2100	1546(40.5)	554(40.2)		
Living area	Rural	4313	3173(83.2)	1140(82.7)	0.171	0.680
	City	878	640(16.8)	238(17.3)		
BMI at the first antenatal visit (kg/m^2^)	<24	4623	3429(89.9)	1194(86.6)	11.188	0.001
	≥24	568	384(10.1)	184(13.4)		
Alcohol consumption	No	5101	3752(98.4)	1349(97.9)	1.513	0.219
	Yes	90	61(1.6)	29(2.1)		
Smoking	No	5170	3802(99.7)	1368(99.3)	4.802	0.028
	Yes	21	11(0.3)	10(0.7)		
Anemia	No	5026	3697(97.0)	1329(96.4)	0.868	0.352
	Yes	165	116(3.0)	49(3.6)		
Virus infection	No	4899	3621(95.0)	1278(92.7)	9.409	0.002
	Yes	292	192(5.0)	100(7.3)		
GDM	No	5119	3762(98.7)	1357(98.5)	0.257	0.612
	Yes	72	51(1.3)	21(1.5)		
History of medical disorders	No	4899	3620(94.9)	1279(92.8)	8.591	0.003
	Yes	292	193(5.1)	99(7.2)		
Conception climate	Winter type	2201	1630(42.7)	571(41.4)	2.717	0.257
	Transitional type	1693	1253(32.9)	440(31.9)		
	Summer type	1297	930(24.4)	367(26.6)		
Infant sex	Boy	2718	1987(52.1)	731(53.0)	0.356	0.551
	Girl	2473	1826(47.9)	647(47.0)		
psychological stress	No	5004	3673(96.4)	1331(96.6)	0.101	0.750
	Yes	184	137(3.6)	47(3.4)		

Abbreviations: IA, induced abortion; BMI, body mass index; GDM, gestational diabetes mellitus. Categorical data was compared using the Chi-square test. *p < 0.05.

### Incidence of HDP among nulliparous women with different maternal characteristics

There are a total of 258 (5.0%) participants diagnosed with HDP, including 141(2.7%) GH and 117(2.3%) PE (Table 2). Number and percentage of HDP,GH and PE by different maternal characteristic were shown in Table 2. Those with age over 35, BMI at first antenatal visit over 24 during pregnancy or history of medical disease were having higher risks of HDP than other different maternal characteristics (P < 0.05). (Table 2) Given that some subgroups of PDH did not have pregnant women who smoked or drank, these factors were not adjusted in the subsequent multivariable logistic analysis.

**Table 2:** Risk of hypertensive disorder of pregnancy by different maternal characteristics in nulliparous women.

Characteristics		Observation population (N)	HDP	GH	PE
			N(%)	N(%)	N(%)
Overall		5191	258(5.0)	141(2.7)	117(2.3)
Maternal age (years)	<35	5138	241(4.7)	130(2.5)	111(2.2)
	≥35	53	17(32.1)	11(20.8)	6(11.3)
Education (years)	≤12	3974	203(5.5)	109(2.7)	94(2.4)
	>12	1217	51(4.5)	32(2.6)	23(1.9)
Occupation	Unemployed	3091	155(5.0)	82(2.7)	73(2.4)
	Employed	2100	103(4.9)	64(2.8)	44(2.1)
Living area	Rural	4313	220(5.1)	123(2.9)	97(2.2)
	City	878	38(4.3)	18(2.1)	20(2.3)
BMI at first antenatal visit (kg/m^2^)	<24	4623	208(4.5)	106(2.3)	102(2.2)
	≥24	568	50(8.8)	35(6.2)	15(2.6)
Alcohol consumption	No	5101	256(5.0)	141(2.8)	115(2.3)
	Yes	90	2(2.2)	0(0.0)	2(2.2)
Smoking	No	5170	257(5.0)	141(2.7)	116(2.3)
	Yes	21	1(4.8)	0(0.0)	1(4.8)
Anemia	No	5026	245(4.9)	131(2.6)	114(2.3)
	Yes	165	13(7.9)	10(6.1)	3(1.8)
Virus infection	No	4899	240(4.9)	131(2.7)	109(2.2)
	Yes	292	18(6.2)	10(3.4)	8(2.7)
GDM	No	5119	251(4.9)	138(2.7)	113(2.2)
	Yes	72	7(9.7)	3(4.2)	4(5.6)
History of medical disorders	No	4899	234(4.8)	127(2.6)	107(2.2)
	Yes	292	24(8.2)	14(4.8)	10(3.4)
Conception climate	Winter type	2201	125(5.7)	69(3.1)	56(2.5)
	Transitional type	1693	82(4.8)	42(2.5)	40(2.4)
	Summer type	1297	51(3.9)	30(2.3)	21(1.6)
Infant sex	Boy	2718	144(5.3)	78(2.9)	66(2.4)
	Girl	2473	114(4.6)	63(2.5)	51(2.1)
psychological stress	No	5004	247(4.9)	135(2.7)	112(2.2)
	Yes	187	11(5.6)	6(3.1)	5(2.6)

Abbreviations: BMI, body mass index; GDM, gestational diabetes mellitus; HDP, hypertensive disorder of pregnancy; GH, gestational hypertension; PE, pre-eclampsia. Categorical data was compared using the Chi-square test. *p < 0.05.

### The incidence of HDP, GH and PE among different number of IA

On further analysis, the number of gravidae with none, one, and two or more previous IAs was 3813 (73.5%), 937 (18.0%), and 441(8.5%), respectively. The incidence of HDP was 5.3%, 4.3% and 3.9% for these three subgroups (p > 0.05). For GH the incidence were 2.8%, 2.5% and 2.5% respectively (p > 0.05). For PE, the respective incidence were 2.5%, 1.8% and 1.4% (p > 0.05). (Table 3) Considering the minimum difference between gravidae having one or two or more IAs, no distinction was made between gravidae with different number of IA in the subsequent analysis.

**Table 3:** The incidence of hypertensive disorders of pregnancy among different number of induced abortion.

Variable		No. Of previous IAs			*p*
		0(N = 3813)	1(N = 937)	≥ 2(N = 441)	
HDP	No	3612(94.7)	897(95.7)	424(96.1)	0.238
	Yes	201(5.3)	40(4.3)	17(3.9)	
GH	No	3706(97.2)	914(97.5)	430(97.5)	0.802
	Yes	107(2.8)	23(2.5)	11(2.5)	
PE	No	3719(97.5)	920(98.2)	435(98.6)	0.203
	Yes	94(2.5)	17(1.8)	6(1.4)	

Abbreviations: IA, induced abortion; HDP, hypertensive disorder of pregnancy; GH, gestational hypertension; PE, pre-eclampsia. Categorical data was compared using the Chi-square test. *p < 0.05.

### The association between inter-pregnancy intervals and risk of HDP among women with previous IA

There were no significant difference in the risk of HDP at different pregnancy intervals (including <12 months, 12–24 months, ≥24 months) in women with prior IA (p > 0.05), but the risk of HDP increased with the prolongation of pregnancy interval. Grouping participants by inter-pregnancy interval (<12 months, 12–24 months, ≥24 months), the number of women with HDP were 2(2.6%), 24(4.0%), 31(4.4%), respectively. However, only 57(4.1%) of women with prior IA developed HDP and among these women only 2(3.5%) occurs during the <12 months inter-pregnancy intervals.

### Association among previous IA and HDP, GH and PE

The risk rates of HDP, GH and PE in the IA group were 4.1%, 2.5% and 1.7%, respectively, which were nominally lower than those in the non-IA group (5.3%, 2.8% and 2.5%, respectively).

However, there was no significantly statistical difference in risk between the two groups. After adjustment for maternal age, body mass index (BMI) at first antenatal visit, education, virus infection and history of medical disorders, previous IA was significantly associated with a lower risk of HDP overall and PE, adjusted odds ratios (aOR^1^) were 0.67(95% CI = 0.49 to 0.91)and 0.61(95% CI = 0.38 to 0.97), respectively, but not with GH (aOR^1^ = 0.73, 95% CI = 0.49 to 1.10). Additional adjustment for occupation, living area, anemia, GDM, psychological stress, conception climate and infant sex, multivariable analysis provided similar results, previous IA was still associated with a lower risk of HDP overall (aOR^2^ = 0.67, 95% CI = 0.49 to 0.91) and PE (aOR^2^ = 0.61, 95% CI = 0.38 to 0.98) (Table 4).

**Table 4:** Association among previous induced abortion and hypertensive disorder of pregnancy.

Variable	Exposed group		Unexposed group		OR(95% CI)	aOR^1^(95% CI)	aOR^2^(95% CI)
	N = 1378	Risk(%)	N = 3813	Risk(%)			
HDP	57	4.1	201	5.3	0.78(0.57–1.05)	0.67(0.49–0.91)*	0.67(0.49–0.91)*
GH	34	2.5	107	2.8	0.88(0.59–1.30)	0.73(0.49–1.10)	0.72(0.48–1.09)
PE	23	1.7	94	2.5	0.67(0.42–1.06)	0.61(0.38–0.97)*	0.61(0.38–0.98)*

Abbreviations: OR, odds ratio; CI, confidence intervals; aOR, adjusted odds ratio; HDP, hypertensive disorder of pregnancy; GH, gestational hypertension; PE, pre-eclampsia; GDM, gestational diabetes mellitus, BMI, body mass index. ^1^Adjustment confounding factors are maternal age, BMI at first antenatal visit, education, history of medical disorders, virus infection. ^2^Adjustment confounding factors are maternal age, education, BMI at first antenatal visit, virus infection, history of medical disorders, occupation, living area, anemia, GDM, psychological stress, conception climate, infant sex. The crude and adjusted OR and 95% CI of HDP, GH and PE were estimated by multivariable logistic regression. *p < 0.05.

## Discussion

In the present study, previous IA was associated with decreased risk of PE among nulliparous women. No protective effect of previous IA on GH has been found. This may be related to the different pathogenesis of GH and PE, although both GH and PE are sub-types of HDP.

The prevalence of IA (26.5%) in our study is lower than the overall population of China (28.95%)^24^. However, it is higher than nulliparous women in Guangzhou (21.96%)^25^. Meanwhile, the prevalence of IA(26.5%) in our study was higher than Western Europe (21%), Northern America (17%) and Oceania (16%), lower than Caribbean (39%), Eastern Europe(38%) and South America(34%)^22^. This variation between populations may be partly attributed to the differences in abortion laws, ethnic background, socioeconomic status. In addition, the study population is primipara in our study, the prevalence of IA is lower than the whole population study, which is also similar to other country, such as Finland^11,26^.

The incidence of HDP(5.0%) in our study is similar to the overall population of China (5.22%)^27^. However, it is higher than pregnant women in Hong Kong (3.2%)^28^. Meanwhile, the incidence of PE (2.3%) in our study was lower than Oceania (2.8–9.2%), Europe (2.8–5.2%) and North America (2.6–4.0%), higher than Africa (0.5–2.3%)^1^. This variation between populations may be partly attributed to the differences in ethnic background, socioeconomic status, and also may related to the decline in abortion rates in developed countries and the increase in primipara with advanced age.

We found that previous IA had protective effect on PE after adjusted possible confounding factors. This findings are consistent with those studies, which reported a reduction in risk of PE after previous IAs^6,7,9–11^. However, there is only a slight difference on protective against PE between gravidae having one versus those with two or more IAs, this is inconsistent with Trogstad^10^ and Parker’s^11^ results that greater reduction in risk of PE after multiple IAs. The reasons for these associations still unclear, previous studies suggested that local injury of the endometrium caused by an IA induces an inflammatory response that promotes successful placentation and consequently reduces the risk of HDP, the reduction in PE risk was greatest among women with inter-pregnancy interval less than 1 year. For as repair processes occur, the immune response subsides, and the benefit of endometrial injury lessens over time^11,29^. In our study, only 3.5% women with prior IA developed HDP within 12 months of the inter-pregnancy intervals. Thus, immune tolerance to paternal antigens in fetal cells after the previous pregnancy may plays an important role in reduction risk of PE^30^. However, A retrospective cohort study conducted by Bhattacharya,S in Scotland, a total of 120033 women with a documented second pregnancy from hospital admissions registry data between 1981 and 2007^19^. They found that previous IA increases the risk of PE and speculated placental position and function could occur due to disruption of the endometrium by vigorous curettage, and then increasing the risk of PE^31^. Although, there were no other studies to support this inference, in order to better understand the protective mechanism of IA on PE, whether placental position and function can reduce the protective effect of IA on PE is worth further exploring.

Our study also found no protective effect of IA on GH, which is keeping the same results as Holmlund S *et al*. reported that previous IA was not associated with GH (OR = 1.07, 95% CI = 0.92 to 1.24^18^. Few related studies have reported, and the mechanism of GH is unclear. Therefore, it is necessary to carry out more relevant research among different races and populations.

The present study is not without limitations. First, we only studied women with fixed sexual partners and did not compare the impact of paternity changing on GH and PE, which however was uncommon in the local Chinese population. Second, in this study, there are few reports of smoking and drinking behavior during pregnancy, so we did not adjust these two factors. Of course, we can not exclude the phenomenon of concealment, but it should have little impact on our findings. Third, not all possible confounding factors have been collected, we only collected some changeable influencing factors, and some unchangeable factors were not collected, such as the family history of hypertension, DM and HDP and some gene variables, which may have a certain impact on our research conclusions. Fourth, lack of information on the date of diagnosis of HDP limited our ability to examine the association by early and later onset PE, which might have different etiologies. Finally, not all before 12 weeks pregnant women can be recruited into the cohort, which will have a certain impact on the representativeness of the study.

Our study also has several strengths in comparison with previous studies of the field. In China, The relationship between abortion and HDP has attracted the attention of scholars, but most of them never differentiate the induced and spontaneous abortions^27,28,31^. Only one study conducted by Chen *et al*.^32^ listed the incidence of PE on surgical abortion women, medical abortions women and primigravies. Therefore, our study is helpful to understand the relationship between previous IA and GH, PE in Chinese population. In addition, the community-based prospective cohort study not only reduce the selection bias but also across a wide range of confounding variables that have not been consistently included in previous studies, such as virus infection, psychological stress, conception climate *etc*.^1,33–35^. Moreover, diagnosis of HDP and its sub-types in our study was based on medical records not self-report, which minimized potential disease misclassification.

Due to ethnic, economic and cultural differences, the incidence of PE and IA in China were different from that in other countries, However, our findings are reasonable in etiology of PE. Therefore, they may have some significance to other nationalities, but further research is needed.

In summary, our study found that the incidence of HDP, GH and PE were 5.1%, 1.7% and 2.6%, respectively. Previous IA was associated with decreased risk of PE among nulliparous women, but not associated with GH. This study also gives us a implication: we should treat IA from different perspectives. Although repeated IA may have adverse effects on maternal and infant health, there may also be advantages, such as reducing the risk of PE.

## Methods

### Data sources and study population

This study is a sub-project of Mother and Child Cohort Study in liuyang, which was designed to study pregnancy complications and pregnancy outcomes. Liuyang is a ‘county-level city’ with 4 city districts and 33 towns. Stratified random sampling was used to select the study participants. According to the urban-rural ratio, and population density and fertility levels, 2 districts and 11 towns were randomly selected in Liuyang city. All nulliparous women who accepted their first prenatal visit less than 12 weeks gestation to local maternity care unit from June 2013 to November 2014 were recruited in this sub-project if they (1) lived in those selected 13 sites, (2) had regular sex partners, (3) were not less than 20 years old, (4) had a history of first-trimester IA or no IA, and (5) were willing to give informed consent. Women were excluded if they (1) had both previous IA and previous spontaneous abortion, (2) had a multiple pregnancy (3) didn’t conceive naturally, and (4) had a uterine malformation. At the last follow-up in November 2015, finally 5191 primipara were included in this study.

### Follow-up and data collection

At enrollment, baseline information was collected for all participants by trained research gynecologists through a face-to-face interview. The questionnaire included (1) maternal basic characteristics (i.e., maternal age, education, occupation, living area, weight and height, smoking and alcohol consumption), (2) reproductive history (i.e., number, time and types of abortion, and pregnancy complications history), (3) chronic disease history(i.e., DM, hypertension, heart disease, nephropathy, pyelonephritis, chronic inflammation of urinary tract, hyperthyroidism, hepatic disease, chronic gastrointestinal diseases, chronic respiratory diseases), and (4) psychological stress. Routine obstetric examinations and laboratory examinations were also conducted afterwards. Then, participants were followed up at 16–20 weeks, 21–24 weeks, 28–36 weeks, 37 weeks of pregnancy to delivery and 4–6 weeks after delivery, and routine obstetric examinations were conducted at each follow-up. Blood pressure status, pregnancy complications and Hepatitis B virus and rubella virus infection status from hospital records were collected after each follow-up. After completion of the information acquisition, the health worker checked the information for completeness and consistency. Supervisors subsequently checked the information as well. Errors and inconsistencies were corrected, if necessary, the information was optimized during the next follow-up. Members of the research group coded and entered the data using double data entry and validation. After the data checks were completed, a logic check and statistical analyses were conducted.

### Variable definition

First-trimester IA refers to an IA that occurred before 12 gestational weeks, including medical and surgical IA. HDP was defined as having either GH or PE. GH is defined as systolic blood pressure of ≥140 mmHg and/or diastolic blood pressure of ≥90 mmHg at ≥20 weeks of gestation^36^. In our study, considering that there are few other cases except GH and PE, therefore, PE, eclampsia(including chronic hypertension with eclampsia), and chronic hypertension superimposed PE (including chronic hypertension with GH) were included into PE group together. PE is characterized by new hypertension, along with proteinuria (300 mg of protein in a 24-hour urine sample or>1+ on dipstick in two urine samples) after 20 weeks of gestation^36^. Eclampsia is the occurrence of a seizure in women in association with PE, in the absence of any other cause for seizures^36^. Chronic hypertension superimposed PE is defined as: (1) chronic hypertension (present at ≥20 weeks of gestation and /or before pregnancy) superimposed on proteinuria at ∼20 weeks of gestation; (2) the deterioration of hypertension and/or proteinuria at ∼20 weeks of gestation in patients with chronic hypertension with proteinuria at ≥20 weeks of gestation^36^. Chronic hypertension superimposed eclampsia is the occurrence of a seizure in women in association with chronic hypertension, in the absence of any other cause for seizures^36^. Psychological stress were assessed using the Pregnancy Pressure Scale (PPS)^37^ at enrollment. The Cronbach’s α of the scale was 0.84. It included 30-items,the factors of the PPS are as follows: factor one is “the sense of stress caused by identifying with the role of parents” (15 items), factor two is “the sense of stress caused by ensuring the health and safety of mother and child” (8 items), factor three is “the sense of stress caused by changes in physical appearance and physical activity” (4 items), and the last three items are classified as other factors (worry about not being able to take good care of their baby, worry that having children will affect the relationship between husband and wife, worry about not being able to provide good living conditions for children). According to the score of self-assessment,the scale score is equal to the total score divided by the number of items. “no” means no pressure, scale score is 0, “yes” means having pressure, scale score is “0.033–3.000”. In Liuyang, pregnant women are routinely screened for GDM using the NDDG criteria during their late 2 nd trimester. The diagnostic criteria are as follows:75 g oral glucose tolerance test(OGTT)test was performed at 24–28 weeks of gestation. The fasting blood glucose (FPG), one hour blood glucose and two hour blood glucose were 5.8 mmol/l, 10.6 mmol/l and 9.2 mmo/l, respectively. GDM is diagnosed if at least 2 of the 3 glucose values in OGTT exceed the preset thresholds^38^. Viral infection included hepatitis B virus infection and rubella virus infection.**“**Yes**”** means viral infection are positive, otherwise it is“No”. “History of medical disorders” means women who had chronic diseases before this pregnancy, including DM, hypertension, heart disease, nephropathy, pyelonephritis, chronic inflammation of urinary tract, hyperthyroidism, hepatic disease(excluding HBV infection), chronic gastrointestinal diseases, chronic respiratory diseases,etc.**“**Yes**”** means one or more of them are positive, otherwise it is “No”. Conception climate was divided into summer type (June to September), winter type (December to February) and transitional type(March to May, and October to November).

### Statistical analyses

Chi-square tests were used to analyze the distributions of maternal characteristics between IA group(exposed) and none IA (unexposed)group. Chi-square tests were also used to analyse the risk of HDP, GH and PE among different maternal characteristics. In order to examine the association of previous IA on the risk of HDP, GH and PE, multivariable logistic regression (odds ratios and 95% confidence intervals) was used to adjust for background variables considered confounders. Potential confounders^1,31,32^ were selected on the basis of previous literature on maternal risk factors on HDP, GH and PE. The original confounders included maternal age, education, occupation, living area, BMI at the first antenatal visit, smoking, alcohol consumption, anemia, virus infection, GDM, history of medical disorders, conception climate, infant sex and psychological stress. Given that some subgroups of PDH did not have pregnant women who smoked or drank, these two factors were not adjusted in the subsequent analysis. The adjustment steps are as follows: First, adjustment was made for maternal age, education, BMI at first antenatal visit, history of medical disorders, virus infection. Second, additional adjustment for occupation, living area, anemia, GDM, psychological stress, conception climate, infant sex. All analyses were conducted using IBM SPSS Statistics version 23.0.

### Ethical considerations

The study was approved by the Ethics Committee of Xiang ya School of Public Health, Central South University (2/24/2011). All the methods in the present study were carried out in accordance with guidelines of the Declaration of Helsinki. All participants provided written informed consent.
